# Pan-cancer analysis from multi-omics data reveals AAMP as an unfavourable prognostic marker

**DOI:** 10.1186/s40001-023-01234-z

**Published:** 2023-07-27

**Authors:** Yang Wang, Ting Liu, Ke Zhang, Rong-hai Huang, Li Jiang

**Affiliations:** 1 Department of General Surgical Department, Beijing Ditan Hospital, Capital Medical University, No. 8 Jing Shun East Street, Chaoyang District, Beijing, 100015 People’s Republic of China; 2 Department of Pathology, Beijing Ditan Hospital, Capital Medical University, No. 8 Jing Shun East Street, Chaoyang District, Beijing, 100015 People’s Republic of China

**Keywords:** AAMP, Pan-cancer, Prognostic, Multi-omics analysis

## Abstract

**Objectives:**

Angio-associated migratory cell protein (AAMP) is a protein that participates in cell migration and is reported to be involved in cancer progression. However, the molecular mechanism of AAMP in pan-cancer is not known.

**Methods:**

We used multi-omics data, such as TIMER, TCGA, GTEx, CPTAC, HPA, and cBioPortal to analyze AAMP expression, and gene alteration in pan-cancer. Univariate Cox regression and Kaplan–Meier were utilized to explore prognostic significance of AAMP expression level. We applied Spearman analysis to investigate the correlation between AAMP and TMB, MSI, immune cell infiltration, immune checkpoints. Moreover, we mainly studied liver hepatocellular carcinoma(LIHC) to explore AAMP expression, clinical significance, and prognosis. Cox regression analysis was used to study independent factor to predict prognosis for AAMP in LIHC. GSEA was utilized to investigate the biological function for AAMP in LIHC.

**Results:**

AAMP was overexpressed in most cancers, and high AAMP expression was associated with worse overall survival (OS), disease-specific survival (DSS), and progress-free interval (PFI) for LIHC and adrenocortical carcinoma (ACC). Moreover, AAMP had the highest mutation frequency in uterine corpus endometrial carcinoma (UCEC). AAMP was correlated with TMB and MSI in esophageal carcinoma (ESCA), stomach adenocarcinoma (STAD), lung squamous cell carcinoma (LUSC), and thyroid carcinoma (THCA). Then, we focus on LIHC to investigate the expression and prognosis of AAMP. AAMP overexpression was related to histological grade and pathological stage in LIHC. Multivariate Cox regression analysis revealed that AAMP overexpression was an independent adverse prognostic marker for LIHC. AAMP expression was correlated with immune cell infiltration and immune checkpoints in LIHC. Function enrichment analysis indicated the participation of AAMP in the cell cycle and DNA replication.

**Conclusions:**

AAMP was a latent prognostic indicator for pan-cancer and had high potential as a biomarker for the diagnosis and prognosis of LIHC.

## Introduction

Cancer is a major cause of death affecting human health worldwide. There are an estimated 19.3 million new cancer cases and almost 10.0 million cancer deaths occurred in 2020 globally [1]. Since the occurrence and development of tumors may be accompanied by different gene alterations, it is an important problem to find biomarkers for the diagnosis of different tumors. Pan-cancer research is to analyze multiple aspects of a large number of tumors and examine the differences in genes in different tumor types, so as to have a fuller understanding of tumors and find therapeutic and diagnostic targets for a variety of tumors.

Angio-associated migratory cell protein (AAMP) was first discovered by Beckner when they screened cell surface proteins related to cell motility in melanoma cells [2]. It belonged to the immunoglobulin superfamily and had homologous domains with cell adhesion molecule proteins NCAM, LFA-2, PECAM, etc. The structural characteristics of AAMP protein suggested that it may be involved in cell adhesion and migration. Subsequently, it was found that AAMP was involved in cancer occurrence and development. For example, AAMP could accelerate the adhesion and proliferation of breast cancer cells, and high AAMP expression had a worse prognosis for breast cancer patients [3, 4]. AAMP interacted with EGFR to enhance the proliferation and drug resistance of non-small cell lung cancer cells [5]. The interaction between AAMP and CDC42 could accelerate non-small cell lung cancer cells' metastasis [6]. However, there are few studies on the AAMP gene expression pattern and latent function in pan-cancer.

To study the latency effect of AAMP in pan-cancer, we analyzed the transcription level of AAMP and its relationship with clinical pathology from multiple public databases. Then, we conducted bioinformatics analysis to investigate the biological function and prognostic significance of AAMP in pan-cancer.

## Materials and methods

### Expression of AAMP in pan-cancer

Tumor Immune Estimation Resource (TIMER) is an online platform for analyzing immune cell infiltration in tumors and gene expression differences between tumors and normal tissues in the TCGA database [7]. We analyzed AAMP expression in pan-cancer from TIMER. Because there were no normal tissues in some tumors from TIMER, so we downloaded the expression profile data of 33 tumors in TCGA and GTEx to compare AAMP gene expression in pan-cancer. The Wilcoxon test was investigated the difference in AAMP gene expression between tumors and normal tissues. AAMP expression in tumors and its matched normal tissues was studied by paired sample *T* test. AAMP expression at the protein level was analyzed using the CPTAC data set from the UALCAN and immunohistochemical image analysis from the HPA database. In addition, the ROC curve was used to estimate the diagnostic significance of AAMP using the pROC package in R. Then, we downloaded RNA sequence and clinical information for liver hepatocellular carcinoma(LIHC) from LIRI-JP in the ICGC database to verify the expression and prognosis of AAMP.

### Prognosis analysis in pan-cancer

According to the median value of AAMP, we separated AAMP expression into AAMP high-expression and low-expression groups. Univariate regressive analysis was used to explore the impact of AAMP on the prognosis of 33 kinds of tumors using a survival package and forest plots for visualization. The prognostic indicators included overall survival (OS), disease-specific survival (DSS), and progress-free interval (PFI). 

### Gene mutation, TMB, and MSI analysis in pan-cancer

Gene mutation is a common mode of epigenetics. cBioPortal is an online website for studying gene mutation analysis in tumors [8, 9]. Gene alteration contains mutation, structural variant, amplification, and deep deletion. Moreover, tumor mutation burden (TMB) and microsatellite instability (MSI) are two highly effective markers for tumor immunotherapy. Tumors with higher TMB can recruit more neoantigens on the surface of tumor cells, increase the immunogenicity of tumors, and improve the efficacy of immunotherapy [10]. Studies have shown that tumors with high MSI are highly sensitive to immune checkpoint inhibitor treatment [11]. Spearman correlation analysis was applied to discuss the correlation between AAMP expression and TMB, MSI.

### Association of AAMP expression with clinicopathological characteristics in LIHC

The clinical information of LIHC patients was acquired from TCGA data, including age, sex, T stage, N stage, M stage, clinical stage, and other clinical features. Moreover, the Chi-Square test was used to study the correlation between AAMP and clinical parameters.

### Relationship of AAMP expression with the prognosis of LIHC and its nomogram

We used the Kaplan–Meier curve to analyze the effect of AAMP expression on OS, PFI, and DSS in LIHC. Moreover, we explored the independent prognostic factors of AAMP in LIHC by univariate and multivariate regression analysis. The 1- year, 3- year, and 5- year survival rate of AAMP in LIHC was investigated using a time ROC curve. A calibration curve was drawn to evaluate the precision accuracy of the nomogram.

### Immune cell infiltration and immune checkpoints analysis in LIHC

We used the ssGSEA algorithm in R packet-GSVA to discuss the correlation of AAMP with 24 kinds of immunocyte infiltration [12, 13]. Moreover, we used the ESTIMATE package in R to estimate the stromal cells and immune cells in tumor tissue, predict tumor microenvironment (TME) by immune and stromal scores, and analyze the association of AAMP with stromal and immune score in LIHC [14]. The presence of immune checkpoint inhibitors has made significant progress in immune therapy. We analyzed the difference in expression of eight common immune checkpoints between the high and low AAMP expression groups to predict the effect of immunotherapy. Tumor Immune Dysfunction and Exclusion (TIDE) algorithm is used to predict the response to cancer immunotherapy. The efficacy of immune checkpoint blockade (ICB) is poorer with a higher TIDE score, and the survival time is shorter after receiving ICB treatment [15, 16]. We predicted immunotherapy response by analyzing TIDE scores of high and low AAMP expression groups in LIHC.

### Gene enrichment analysis in LIHC

The AAMP was classified into high- and low-expression groups according to the median expression. We explored the DEGs between high- and low-expression groups in LIHC using the DESeq2 (version 1.26.0) package. Gene Ontology (GO) enrichment and GSEA were performed to explore the relevant pathways involved in AAMP in LIHC using the “Cluster Profiler” package of R language. GO enrichment contains molecular function (MF), cellular component (CC), and biological process (BP). *P* < 0.05,  and FDR < 0.25 are defined as significantly enriched.

### Statistical analysis

Wilcoxon test was utilized to analyze the differential expression of two groups. The chi-square test was explored the association between AAMP expression and clinical features. Univariate regression analysis and the Kaplan–Meier curve were used to investigate the effect of AAMP on prognosis. We predicted the impact of AAMP expression in LIHC on 1-, 3-, and 5-year's OS by timeROC package. Spearman correlation analysis was used to research the correlation of AAMP expression with TME and immune checkpoints.

## Results

### AAMP expression analysis and diagnostic value in pan-cancer

**Fig. 1:**
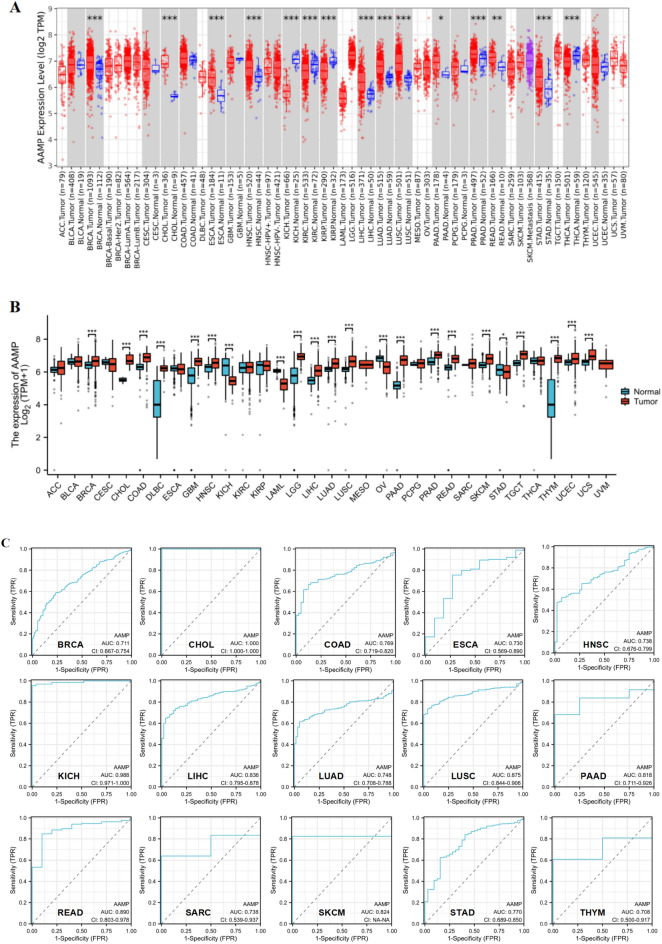
AAMP gene expression level in tumors and normal tissues and diagnostic value. **A** AAMP expression in tumors and normal tissues from TIMER database. **B** AAMP expression in tumors and normal tissues from TCGA and GTEx database. **C** ROC curve for AAMP in pan-cancer. (*P < 0.05, **P < 0.01, ***P < 0.001)

TIMER results demonstrated that AAMP expression was highly expressed in BRCA, CHOL, ESCA, HNSC, LIHC, LUAD, LUSC, PAAD, PRAD, READ, and STAD while lowly expressed in KICH, KIRC, KIRP, and THCA (Fig. 1A). In TCGA, because some tumors lack normal samples, we combined TCGA with GTEx to analyze AAMP expression in tumors and normal tissues. We found that AAMP expression was overexpressed in 18 tumors, while AAMP was lowly expressed in KICH, LAML, OV, and STAD (Fig. 1B). The ROC curve was used to evaluate the diagnostic value of AAMP in pan-cancer. The results demonstrated that AAMP had a certain accuracy (AUC > 0.7) in predicting 15 tumors, including BRCA, CHOL, COAD, ESCA, HNSC, KICH, LIHC, LUAD, LUSC, PAAD, READ, SARC, SKCM, STAD, and THYM.

### Prognostic analysis in pan-cancer

**Fig. 2:**
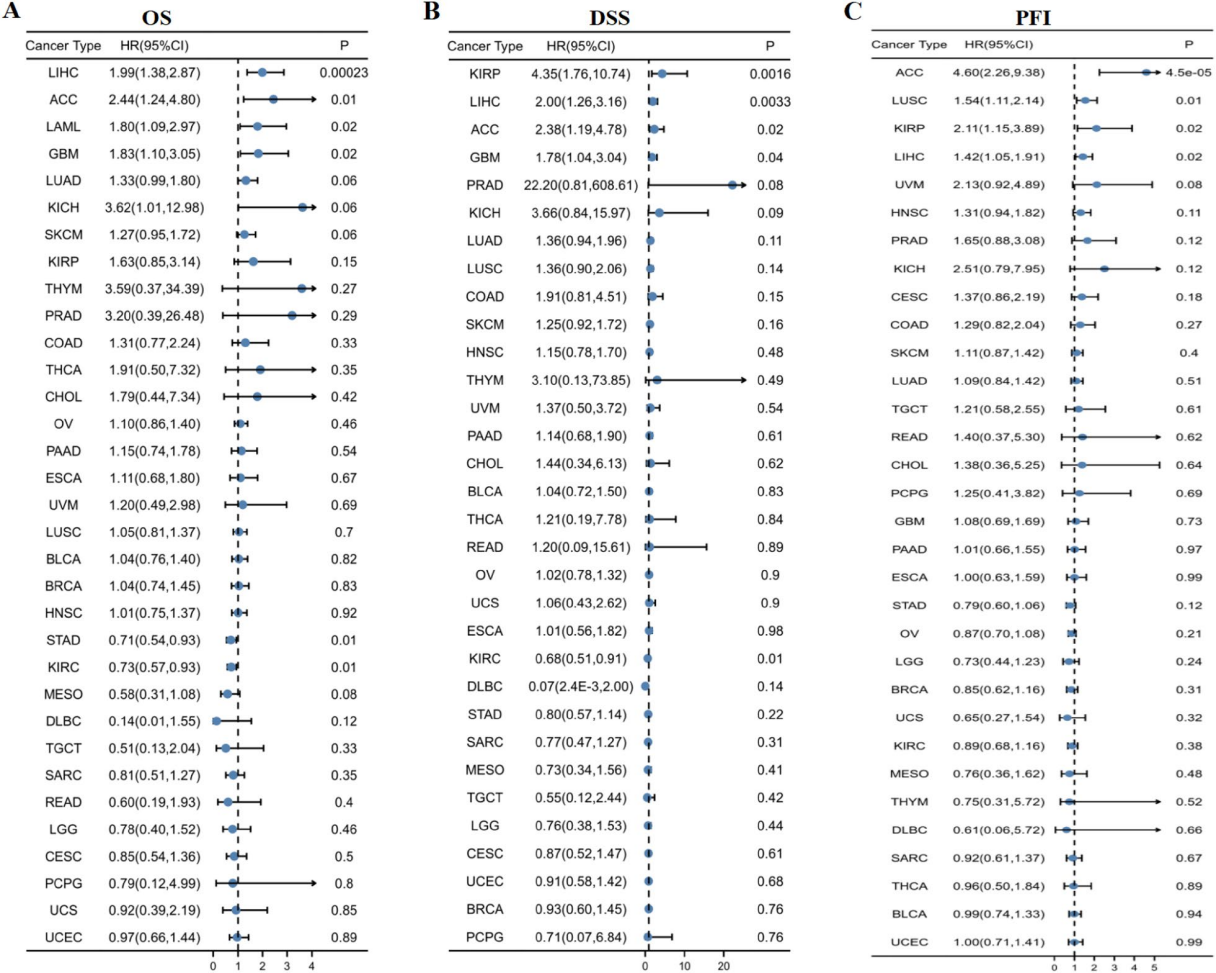
Prognosis of AAMP in TCGA for pan-cancer. **A** Overall survival (OS) of AAMP. **B** Disease-specific survival (DSS)of AAMP. **C** Progress-free interval (PFI) of AAMP

We discussed the prognosis of patients with LIHC in the high- and low-expression groups of AAMP. The results of OS in pan-cancer demonstrated that the expression of AAMP correlated with the OS for LIHC, ACC, LAML, GBM, STAD, and KIRC. The high expression of AAMP had poorer OS in LIHC, ACC, LAML, and GBM and better OS in STAD and KIRC (Fig. 2A). Regarding DSS, AAMP was a risk factor in KIRP, LIHC, ACC, and GBM and a protective factor in KIRC (Fig. 2B). In terms of PFI, overexpression AAMP in ACC, LUSC, KIRP, and LIHC had poor PFI (Fig. 2C). The highly expressed AAMP had inferior OS, DSS, and PFI in ACC and LIHC.

### Gene mutation, TMB, and MSI analysis of AAMP in pan-cancer

**Fig. 3:**
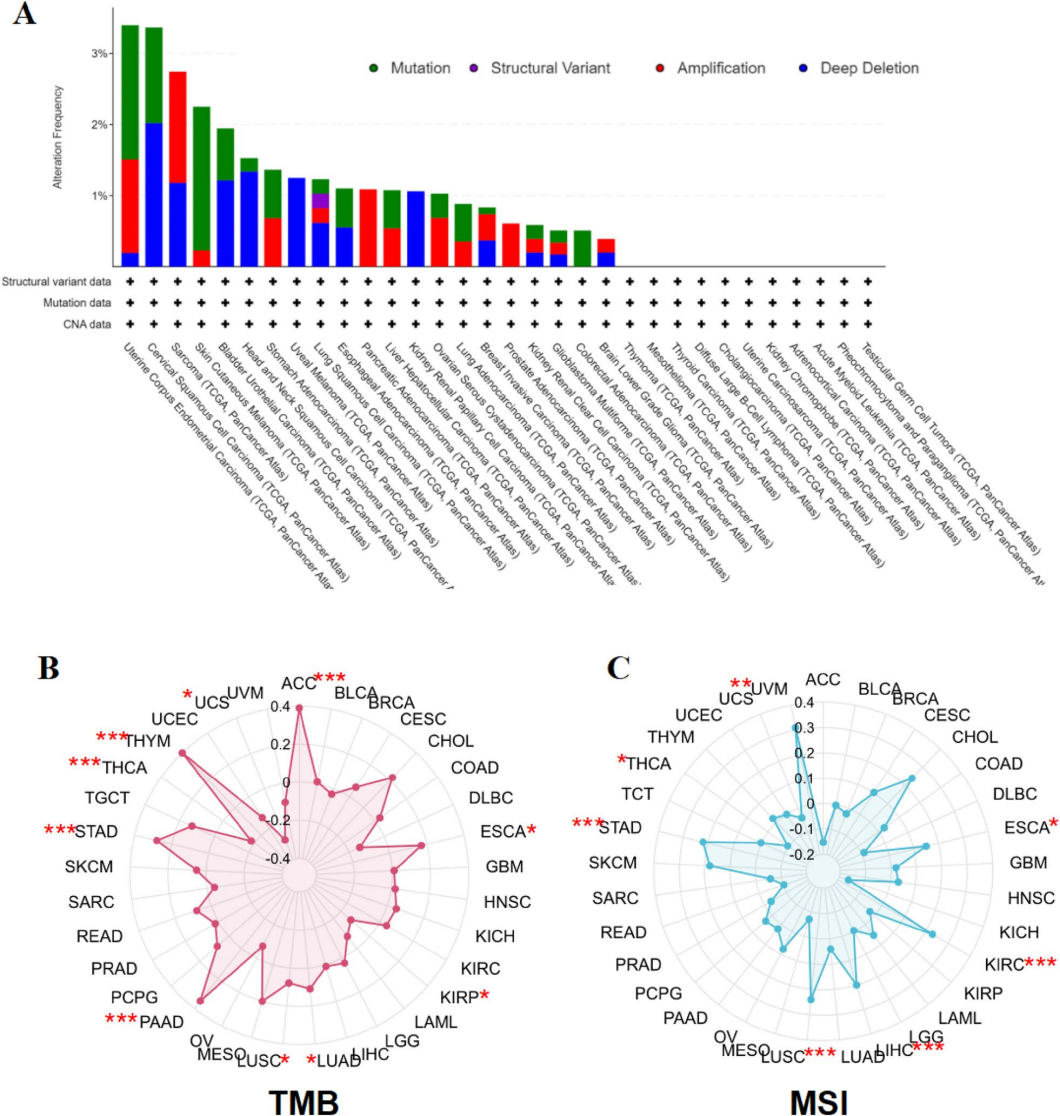
Gene alteration, TMB, and MSI analysis of AAMP. **A** Gene alteration for AAMP in cBioPortal database. **B** Correlation of AAMP with TMB. **C** Correlation of AAMP with MSI. (*P < 0.05, **P < 0.01, ***P < 0.001)

cBioPortal was used to research AAMP gene alteration in pan-cancer. The highest frequency of gene alterations was found in UCEC(mutation 1.89%, amplification 1.32%, deep deletion 0.19%), followed by CESC (mutation 1.35%, deep deletion 2.02%) and SARC(amplification1.57%, deep deletion 1.18%) (Fig. 3A). The results of the TMB correlation analysis exhibited that AAMP expression had a positive correlation with TMB in ACC, ESCA, LUAD, STAD, LUSC, THYM, and PAAD while negative correlation in KIRP, THCA, and UCS (Fig. 3B). As for MSI, AAMP was positively related to MSI in ESCA, STAD, KIRC, LUSC, LIHC, and UVM, and negatively in THCA (Fig. 3C).

### Expression of AAMP and the relationship between AAMP expression and clinical pathology in LIHC

**Fig. 4:**
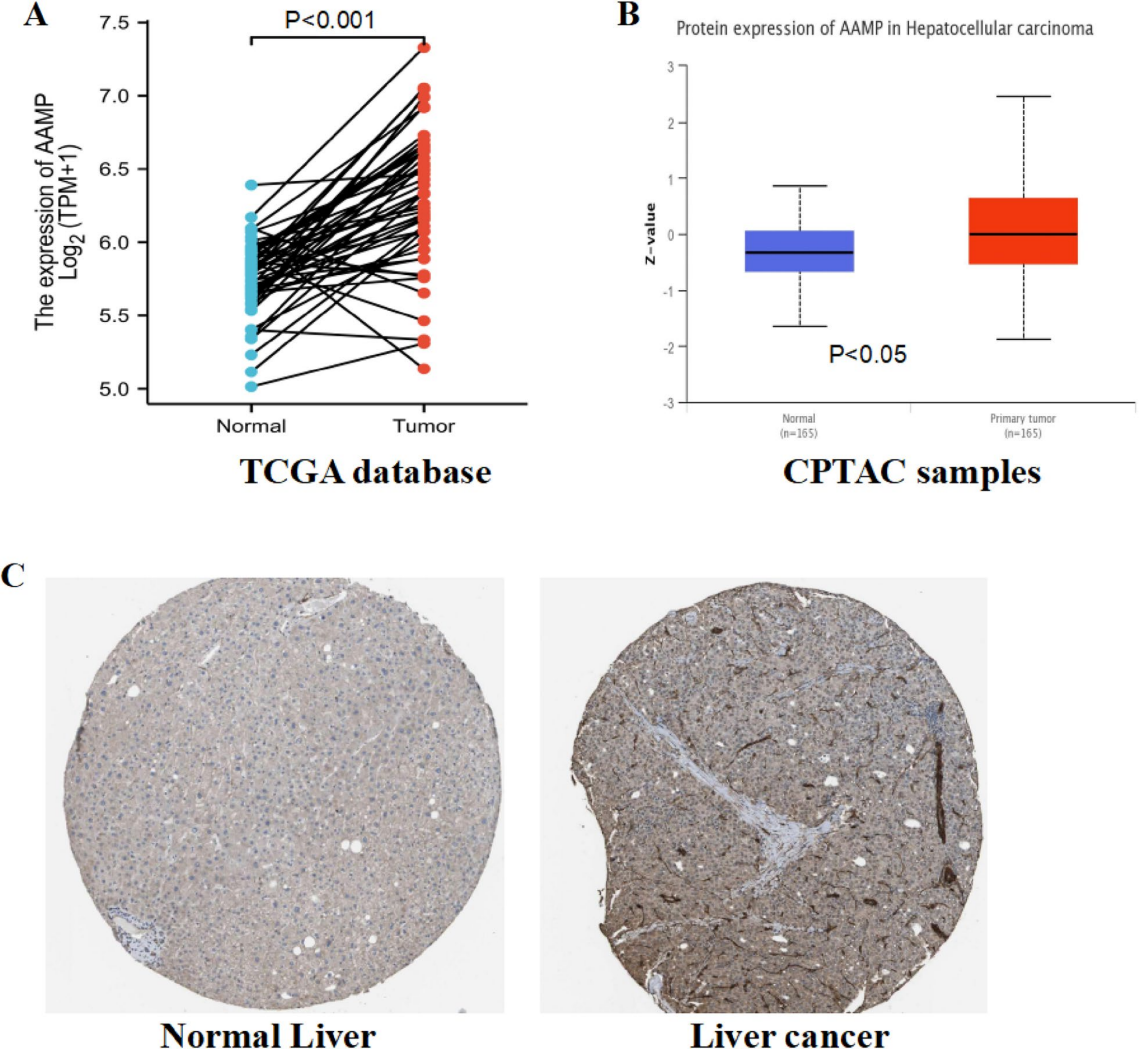
Expression of AAMP in different databases. **A** AAMP expression in liver cancer and matched normal tissues. **B** AAMP protein expression in liver cancer and normal samples in CPTAC database. **C** Expression of AAMP in liver cancer and normal tissue in HPA database

AAMP had higher expression in LIHC than in matched normal tissues (Fig. 4A). Furthermore, the CPTAC data set in the UALCAN database revealed that AAMP protein expression had over-expression in LIHC (Fig. 4B). The immunohistochemical staining also confirmed the increased expression of AAMP in liver cancer (Fig. 4C).

Meanwhile, we acquired the clinical characteristics of LIHC from the TCGA database, and our results revealed that AAMP expression was related to histological grade and pathological stage of LIHC. The high expression rate of AAMP (G3 + G4,80/136) in LIHC with a high histological grade was higher than that with a low histological grade (G1 + G2,105/233). The increased expression rate of AAMP (55/90) in advanced HCC (Stage III + IV) was higher than that in early LIHC (Stage I + II) (120/260) (Table 1). These results indicated that the expression of AAMP promoted LIHC progression.

**Table 1:** Correlation between AAMP expression and clinical characteristics in TCGA database

Characteristics	Low expression of AAMP	High expression of AAMP	*P* value
*n*	187	187	
Age			0.570
< = 60	86	91	
> 60	101	95	
Gender			0.740
Female	59	62	
Male	128	125	
Pathologic T stage			0.052
T1&T2	146	132	
T3&T4	38	55	
Pathologic N stage			0.693
N0	121	133	
N1	1	3	
Pathologic M stage			1.000
M0	133	135	
M1	2	2	
Histologic grade			0.011*
G1&G2	128	105	
G4&G3	56	80	
Pathologic stage			0.014*
Stage I&Stage II	140	120	
Stage III&Stage IV	35	55	

*P < 0.05

### Prognosis and nomogram of AAMP in LIHC

**Fig. 5:**
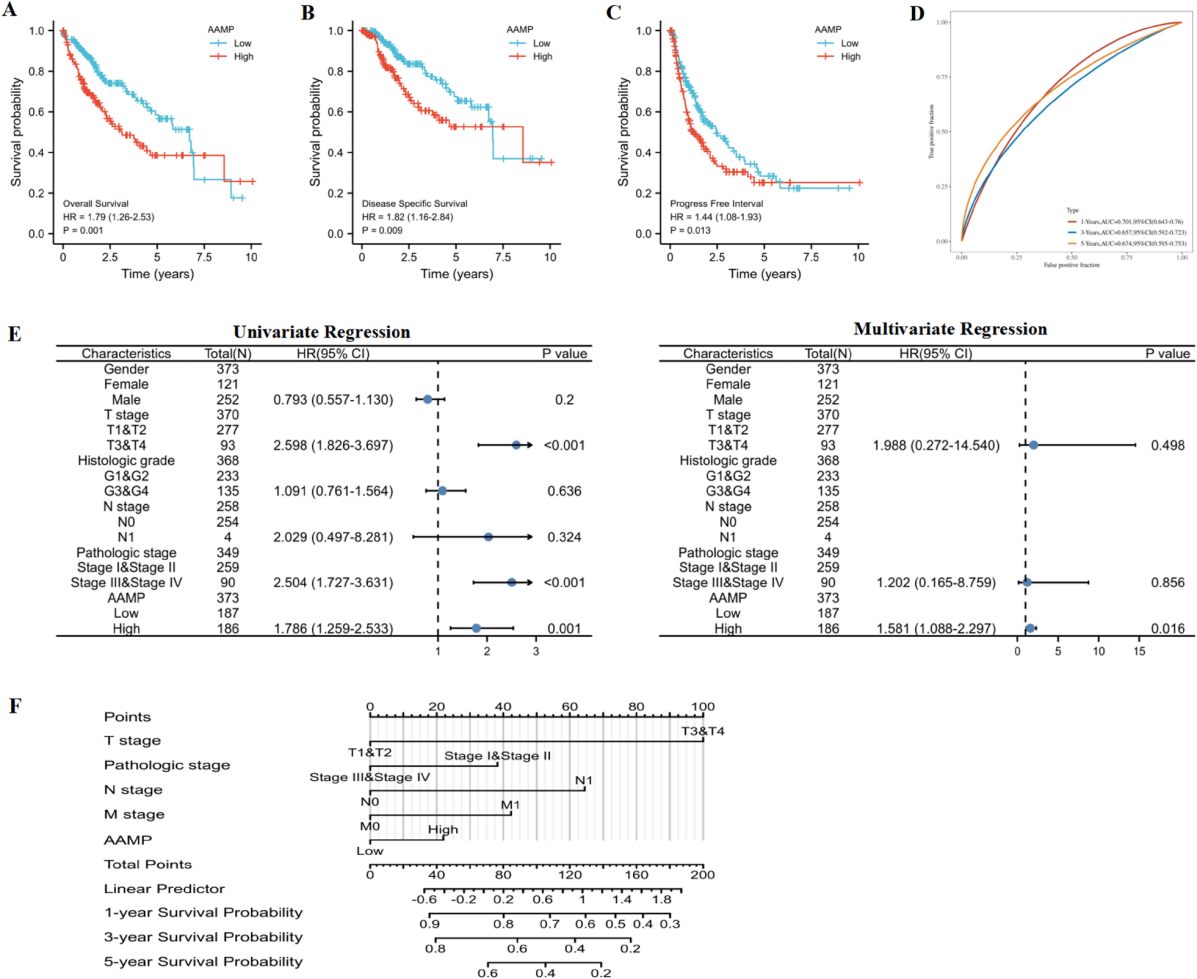
Prognosis and nomogram of AAMP in LIHC. **A** Kaplan–Meier survival curve of AAMP for OS. **B** Kaplan–Meier survival curve of AAMP for DSS. **C** Kaplan–Meier survival curve of AAMP for PFI. **D** ROC survival curve of 1, 3, and 5 year OS. **E** Univariate and multivariate regression analyses of AAMP. **F** Nomogram predicts the risk of progression in patients with LIHC

Kaplan–Meier survival curve also revealed that in LIHC, AAMP in the higher expression group had shorter OS, DSS, and PFI than in the lower expression group (Fig. 5A,B,C). Moreover, 1-, 3-, and 5-year OS was 0.701, 0.657, and 0.674, respectively (Fig. 5D). The univariate and multivariate analysis results considered AAMP expression as an independent prognostic marker in LIHC (Fig. 5E). A nomogram was constructed to predict the 1, 3, and 5 year OS for LIHC (Fig. 5F).

### Verify the expression and prognosis of AAMP in LIHC from ICGC

**Fig. 6:**
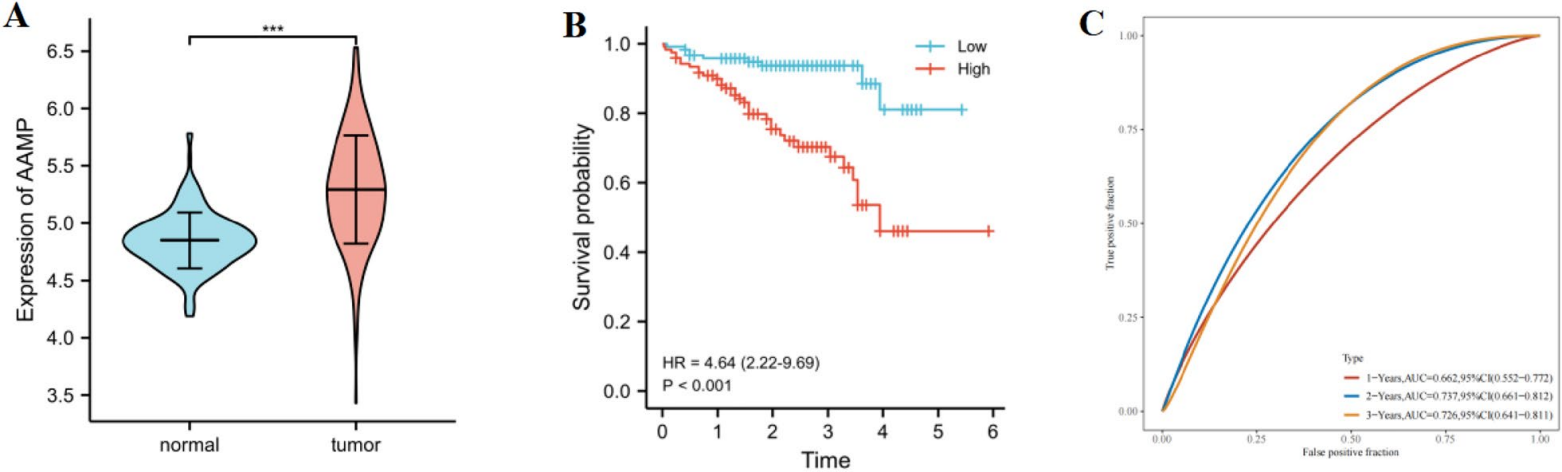
Expression and prognostic analysis of AAMP in the ICGC database. **A** mRNA expression of AAMP in LIHC and normal tissues. **B** Kaplan–Meier survival curve of AAMP in ICGC. **C** ROC survival curve of 1-, 2-, and 3- year OS in ICGC.

To verify the expression and prognosis of AAMP in LIHC, we acquired AAMP gene expression from ICGC. We found that AAMP had higher expression in LIHC than in normal liver tissue, and AAMP overexpression had an adverse outcome in LIHC (Fig. 6A, B). These results were consistent with the TCGA database. The time ROC curve showed that the OS for 1-, 2- and 3-years was 0.662, 0.737, and 0.726, respectively (Fig. 6C). These findings showed that AAMP had an excellent prediction ability in TCGA and ICGC.

### Relevance analysis of AAMP with immunocyte infiltration and immune checkpoints in LIHC

**Fig. 7:**
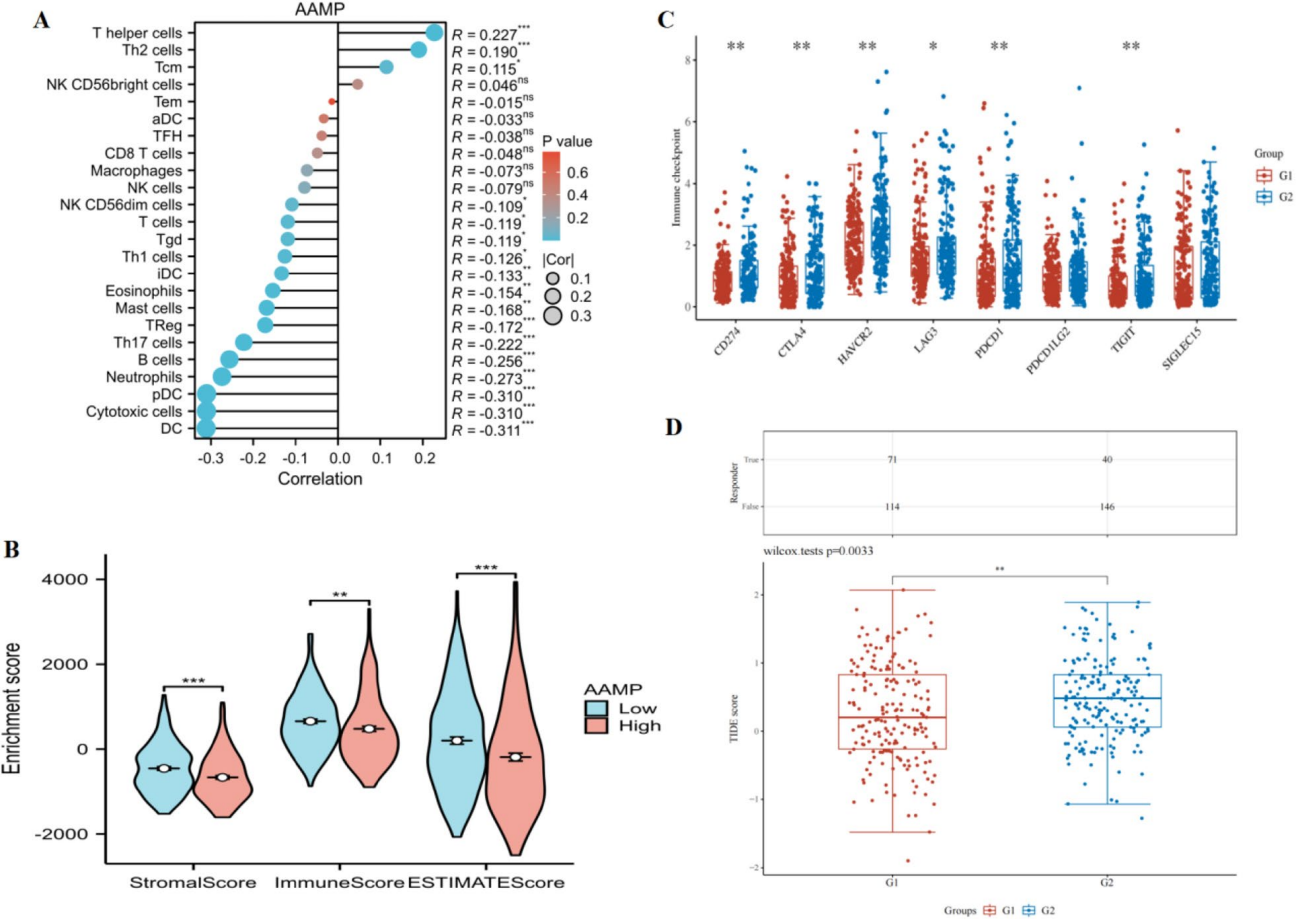
Relationship between the immune microenvironment and AAMP expression in LIHC**.** **A** ssGSEA analysis of AAMP in high and low expression groups. **B** ESTIMATE analysis of AAMP in high and low expression groups. **C** Differential expression of checkpoints in high and low AAMP expression groups. **D** TIDE score in high and low AAMP expression groups. (G1: low expression group, G2: high expression group,*P < 0.05,**P < 0.01,***P < 0.001)

The ssGSEA algorithm showed that AAMP in LIHC was positively correlated with T helper cells, Th2 cells, and Tcm, and negatively linked with DC, Cytotoxic cells, pDC, neutrophils, B cells, Th17 cells, Treg, mast cells, eosinophils, iDC, Th1 cells, Tgd, T cells, NK CD56 dim cells. Among them, AAMP had the highest positive correlation coefficient with T helper cells and the highest negative correlation coefficient with DC (Fig. 7A). The results of the ESTIMATE algorithm demonstrated that the stromal score, immune score, and estimate score of AAMP in the high-expression group were lower than in the low-expression group, which showed that AAMP influenced cancer development through immune cell infiltration (Fig. 7B). Immune checkpoints are closely linked with tumor proliferation, invasion, metastasis, and prognosis of patients, so they are good targets for tumor treatment. The immune checkpoints can activate the intracellular signal pathway to promote the immune response and the escape of tumor cells [17, 18]. The expression of CD274, CTLA4, HAVCR2, LAG3, PDCD1, and TIGIT in the high AAMP expression was higher than in the low AAMP expression group, which showed that AAMP expression was critical immunotherapeutic targets in LIHC (Fig. 7C). In our study, AAMP in high expression had a higher TIDE score and a lower response rate to immunotherapy (Fig. 7D).

### Gene set enrichment analysis

**Fig. 8:**
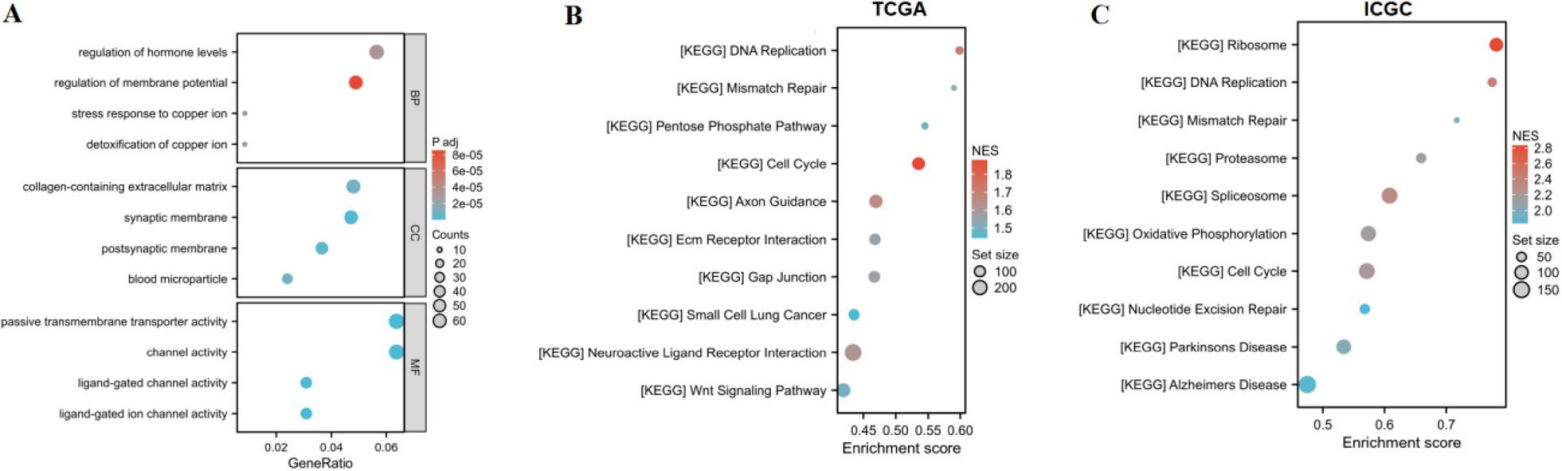
Function enrichment of AAMP in high and low expression groups. **A** GO enrichment analysis of AAMP in high and low expression groups. **B** KEGG of GSEA enrichment for high and low AAMP expression in TCGA database. **C** KEGG of GSEA enrichment for high and low AAMP expression in ICGC database

We conducted an enrichment analysis to elucidate the biological mechanism of AAMP involvement in LIHC. The BP of GO enrichment showed that AAMP was involved in regulation of hormone levels, regulation of membrane potential, stress response to copper ion, and detoxification of copper ion. The results of CC in GO suggested that AAMP participated in collagen-containing extracellular matrix, synaptic membrane, postsynaptic membrane, and blood microparticle. As for MF, AAMP mainly focused on passive transmembrane transporter activity, channel activity, ligand-gated channel activity, and ligand-gated ion channel activity (Fig. 8A). KEGG database from GSEA enrichment showed that high expression of AAMP participated in the cell cycle, DNA replication, axon guidance, neuroactive ligandreceptor interaction, gap junction, ECM receptor interaction, and mismatch repair in TCGA database (Fig. 8B). Furthermore, ribosome, DNA replication, spliceosome, cell cycle, mismatch repair, oxidative phosphorylation, and proteasome were enriched in the high AAMP expression in ICGC database (Fig. 8C). In short, in the two gene sets, the highly expressed AAMP was mainly enriched in cell cycle, DNA replication, and mismatch repair, which participated in the progress of LIHC.

## Discussion

AAMP is a member of the immunoglobulin superfamily and is widely distributed in various types of cells. It is essential in transcriptional activation, cell cycle regulation, protein–protein interaction, and signal transduction [19–21]. AAMP is expressed in a variety of cell types, including endothelial cells, aortic smooth muscle cells, dermal fibroblasts, renal proximal tubular cells, glomerular mesangial cells, human breast cancer cells, human melanoma cells and prostate cancer cells [22–24]. Recent studies have shown that AAMP mainly locates in the cytoplasm and membrane of vascular endothelial cells, affecting the angiogenesis, diffusion, migration, and cytoskeleton remodeling processes of endothelial cells [25, 26]. In addition, it has been reported that AAMP is abnormally up-regulated in metastatic CRC and boosts the occurrence of colorectal cancer by inhibiting SMURF2-mediated RhoA liquefaction and degradation [27]. Furthermore, AAMP is highly expressed in invasive gastrointestinal and breast carcinoma cells and is a marker of poor prognosis [4, 28]. In addition, AAMP plays a positive role in angiogenesis and is regulated by Astrocytes in coculture [29]. These results showed that AAMP is vital in cancer occurrence and progression.

Our study explored AAMP expression, prognostic value, gene alteration, TMB, and MSI in pan-cancer through multiple databases. AAMP had high expression in 18 kinds of tumors, while AAMP had low expression in four tumors. The highly expressed AAMP had inferior OS, DSS, and PFI in ACC and LIHC. We used cBioPortal to study AAMP gene alteration in pan-cancer. In UCEC, AAMP had the highest gene alteration frequency, including mutation 1.89%, amplification 1.32%, and deep deletion 0.19%. TMB and MSI are considered as two biomarkers of response to immunotherapy. Tumors with high TMB and high MSI are sensitive to immunotherapy response [30]. Therefore, we study the correlation between AAMP and TMB, MSI in pan-cancer. AAMP expression was positively correlated with TMB for ACC, ESCA, LUAD, STAD, LUSC, THYM, and PAAD while negatively correlated for KIRP, THCA, and UCS. Regarding MSI, AAMP had a positive relation to MSI in ESCA, STAD, KIRC, LUSC, LIHC, and UVM, and negative relation in THCA. AAMP correlated with TMB and MSI in ESCA, STAD, and LUSC. It can be speculated that in ESCA, STAD, and LUSC, tumors with high expression of AAMP have better responses to immunotherapy.

We focused on LIHC after screening and discussed the AAMP expression, prognosis, clinical features, and immunity. AAMP was over-expressed at the mRNA expression level from the TCGA database and protein expression level from CPTAC. The immunohistochemical staining in the HPA database also confirmed AAMP's high expression in LIHC. Moreover, AAMP overexpression was correlated with histological grade and pathological stage of LIHC. AAMP had higher expression in the higher histological grade and advanced pathological stage. Kaplan–Meier survival curve demonstrated that AAMP's high expression was related to unfavorable OS, DSS, and PFI. The univariate and multivariate analysis results showed that AAMP was an independent adverse prognostic factor for LIHC patients. Furthermore, ROC curve analysis demonstrated that OS of 1, 3 and 5 years was more than 0.6. We also confirmed the high expression of AAMP in LIHC in another different database. The results showed that 1, 2 and 3 year OS was 0.662, 0.737, and 0.726 by ROC curve analysis. These findings suggested that AAMP expression had an excellent prediction ability in TCGA and ICGC and could predict LIHC prognosis, supporting AAMP expression as a new predictor of survival for LIHC.

TME is essential in tumor development and is closely related to patient outcomes [31]. In recent years, increasing studies have confirmed that different TME of patients is vital in mediating late metastasis, immune escape, and immunosuppression. We used the ssGSEA algorithm to explore the association between AAMP and immune cell infiltration in LIHC. With the increase of AAMP expression, the number of DC, Cytotoxic cells, pDC, neutrophils, B cells, Th17 cells, Treg, mast cells, eosinophils, iDC, Th1 cells, Tgd, T cells, NK CD56 dim cells decreased, and the number of T helper cells, Th2 cells, and Tcm increased. Therefore, changes in AAMP expression lead to changes in Th1/Th2. The immunosuppressive state will affect the body's anti-tumor immunity and ultimately result in tumors [32]. Immune and stromal cells, as the non-tumor components of TME, have gradually attracted the attention of researchers due to their essential roles in tumor genesis, metastasis, drug resistance, and prognosis [33, 34]. We found that the stromal score and immune score of AAMP over-expression were lower than AAMP low-expression, indicating that AAMP affected the occurrence and development of LIHC through immunocyte infiltration. Recently, the treatment of advanced malignant patients has been revolutionized by the introduction of immune checkpoint inhibitors [35]. Moreover, common checkpoints included CD274, CTLA4, HAVCR2, LAG3, PDCD1, PDCD1LG2, TIGIT, and SIGLEC15. The expression of CD274, CTLA4, HAVCR2, LAG3, PDCD1, and TIGIT in the AAMP high-expression group was higher than AAMP low expression group, which showed that AAMP was the vital immunotherapeutic target in LIHC. TIDE is considered an indicator of cancer's immune intelligence response rate. The patients with higher TIDE scores have lower immune responses. In our study, the patients with increased expression of AAMP have low immune response rates and cannot benefit from immune checkpoint inhibitors. This may be because high AAMP expression induces a decrease in B and T cells, while low lymphocyte counts indicate a poor host anti-tumor immune response [36].

To further investigate the biological process of AAMP in LIHC, we analyzed GSEA in TCGA and ICGC databases. Interestingly, the highly expressed AAMP in the two databases is mainly concentrated in cell cycle, DNA replication, and mismatch repair, indicating that AAMP causes liver cancer through the above pathways.

We discussed the expression pattern and prognostic significance of AAMP in pan-cancer and LIHC from a bioinformatics perspective, providing a basis for further research on the mechanism of AAMP for LIHC. However, our study had some limitations. We studied AAMP expression in pan-cancer and LIHC only by bioinformatics. Furthermore, many experiments to explore the mechanism of AAMP for LIHC will help developing a more accurate prognosis model for patients and providing a basis for more personalized treatment.

## Conclusion

AAMP is a better molecular marker with diagnostic and prognostic value in pan-cancer, especially in LIHC. High expression of AAMP is significantly associated with poor prognosis of LIHC and is considered an independent prognostic marker by Cox regression. This study provides a theoretical basis for more comprehensive analysis of the clinical application of this molecule in tumor therapy in the future.

## Data Availability

The original manuscript contained in the research report is included in the article. Further inquiries can be made directly to the corresponding author.

## Abbreviations

ACC: Adrenocortical carcinoma
BLCA: Bladder urothelial carcinoma
BRCA: Breast invasive carcinoma
CESC: Cervical squamous cell carcinoma and endocervical adenocarcinoma
CHOL: Cholangio carcinoma
COAD: Colon adenocarcinoma
DLBC: Lymphoid neoplasm diffuse large B-cell lymphoma
ESCA: Esophageal carcinoma
GBM: Glioblastoma multiforme
HNSC: Head and neck squamous cell carcinoma
KICH: Kidney chromophobe
KIRC: Kidney renal clear cell carcinoma
KIRP: Kidney renal papillary cell carcinoma
LAML: Acute myeloid leukemia
LGG: Brain lower grade glioma
LIHC: Liver hepatocellular carcinoma
LUAD: Lung adenocarcinoma
LUSC: Lung squamous cell carcinoma
MESO: Mesothelioma
OV: Ovarian serous cystadenocarcinoma
PAAD: Pancreatic adenocarcinoma
PCPG: Pheochromocytoma and paraganglioma
PRAD: Prostate adenocarcinoma
READ: Rectum adenocarcinoma
SARC: Sarcoma
SKCM: Skin cutaneous melanoma
STAD: Stomach adenocarcinoma
TGCT: Testicular germ cell tumors
THCA: Thyroid carcinoma
THYM: Thymoma
UCEC: Uterine corpus endometrial carcinoma
UCS: Uterine carcinosarcoma
UVM: Uveal melanoma
